# Honey Bee *Apis mellifera* Parasites in the Absence of *Nosema ceranae* Fungi and *Varroa destructor* Mites

**DOI:** 10.1371/journal.pone.0098599

**Published:** 2014-06-23

**Authors:** Dave Shutler, Krista Head, Karen L. Burgher-MacLellan, Megan J. Colwell, Abby L. Levitt, Nancy Ostiguy, Geoffrey R. Williams

**Affiliations:** 1 Department of Biology, Acadia University, Wolfville, Nova Scotia, Canada; 2 Agri-Foods Branch, Forestry and Agri-Foods Agency, Department of Natural Resources, Government of Newfoundland and Labrador, Corner Brook, Newfoundland and Labrador, Canada; 3 Atlantic Food and Horticulture Research Centre, Agriculture and Agri-Food Canada, Kentville, Nova Scotia, Canada; 4 Department of Entomology, Pennsylvania State University, University Park, Pennsylvania, United States of America; 5 Department of Biology, Dalhousie University, Halifax, Nova Scotia, Canada; 6 Institute of Bee Health, Vetsuisse Faculty, University of Bern, Bern, Switzerland; 7 Agroscope, Swiss Bee Research Centre, Bern, Switzerland; University of British Columbia, Canada

## Abstract

Few areas of the world have western honey bee (*Apis mellifera*) colonies that are free of invasive parasites *Nosema ceranae* (fungi) and *Varroa destructor* (mites). Particularly detrimental is *V. destructor*; in addition to feeding on host haemolymph, these mites are important vectors of several viruses that are further implicated as contributors to honey bee mortality around the world. Thus, the biogeography and attendant consequences of viral communities in the absence of *V. destructor* are of significant interest. The island of Newfoundland, Province of Newfoundland and Labrador, Canada, is free of *V. destructor*; the absence of *N. ceranae* has not been confirmed. Of 55 Newfoundland colonies inspected visually for their strength and six signs of disease, only K-wing had prevalence above 5% (40/55 colonies = 72.7%). Similar to an earlier study, screenings again confirmed the absence of *V. destructor*, small hive beetles *Aethina tumida* (Murray), tracheal mites *Acarapis woodi* (Rennie), and *Tropilaelaps* spp. ectoparasitic mites. Of a subset of 23 colonies screened molecularly for viruses, none had Israeli acute paralysis virus, Kashmir bee virus, or sacbrood virus. Sixteen of 23 colonies (70.0%) were positive for black queen cell virus, and 21 (91.3%) had some evidence for deformed wing virus. No *N. ceranae* was detected in molecular screens of 55 colonies, although it is possible extremely low intensity infections exist; the more familiar *N. apis* was found in 53 colonies (96.4%). Under these conditions, K-wing was associated (positively) with colony strength; however, viruses and *N. apis* were not. Furthermore, black queen cell virus was positively and negatively associated with K-wing and deformed wing virus, respectively. Newfoundland honey bee colonies are thus free of several invasive parasites that plague operations in other parts of the world, and they provide a unique research arena to study independent pathology of the parasites that are present.

## Introduction

The study of species invasions has a long history [1], with multiple examples of negative consequences for established organisms, particularly in the case of introduced parasitic diseases [2]–[6]. Many invasions of parasite species arose because of international movement of livestock and agricultural products; collectively the biogeography of the planet is becoming increasingly homogenized. For example, as the beekeeping industry became more industrialized, moving Western honey bees (*Apis mellifera* L.; hereafter honey bees) within and among continents became common, leading to global homogenization of their parasite communities [7]. To illustrate, in only a few years after arrival of parasitic *Varroa destructor* (Anderson and Trueman) mites in eastern North America in 1987 [8], the majority of the continent was reporting their presence. The same scenario played out following introduction of the microsporidian fungus *Nosema ceranae* (Fries) to Europe and then other parts of the world [9]–[13]. As a result of these invasive parasites, heavy economic penalties have been experienced by the honey bee industry via colony losses and reduced productivity of surviving colonies [14]–[16]; both threaten global food security because of reduced pollination services to agriculture [17], [18]. The extent to which these detriments are attributable to specific parasite species is difficult to assess because of concomitant invasion and occurrence of multiple parasites (“parasite” here subsumes viruses, bacteria, and fungi [19]) within colonies, and because of potential interactions among parasites [20], [21]. Moreover, it is difficult to evaluate background historical levels of many important parasites because most screenings postdate their arrival, but see [22]–[24]. Here, in an area free of *V. destructor*, we tested molecularly for presence of viruses and *N. ceranae*, and screened visually for other arthropod parasites. We relate those data to metrics of colony strength.

Among the most detrimental parasites to honey bees is *V. destructor* [21]; the importance of *N. ceranae* is debated [25]–[31]. Several viruses are vectored, and may have their virulence enhanced, by *V. destructor* [32]–[35]. For a variety of reasons, the island of Newfoundland, Canada has thus far purportedly remained free of *N. ceranae*, *V. destructor*, and many common arthropod parasites of honey bees [36]. Important contributors to these parasites' absence include spatial isolation, a small number of beekeepers that can be easily regulated and educated, and import restrictions. As a consequence, Newfoundland colonies offer opportunities for comparisons with colonies currently plagued by a more diverse community of parasites. Moreover, Newfoundland presents the possibility of characterizing the biogeography of a suite of viruses that currently predate invasion of potential vectors.

We evaluated several metrics of colony strength, assessed colonies visually for signs of disease and arthropod parasites, and tested molecularly for presence of five viruses and *Nosema* in honey bee colonies against this backdrop of a relatively rarefied parasite community. Because of *V. destructor*'s role in vectoring viruses and enhancing signs of virus, we predicted that some relatively ubiquitous viruses would be absent, and signs of viruses in Newfoundland honey bees would be less frequent than in areas where *V. destructor* mites occur [35].

## Methods

### Ethics statement

No animal use protocol was required by Acadia University's Animal Care Committee (participant of the Canadian Council on Animal Care) to perform this research on honey bees. Privately owned land was accessed only after obtaining permission from landowners.

### General methods and visual assessments

Within the province of Newfoundland and Labrador, Canada, only the island of Newfoundland is temperate enough to maintain economically viable commercial honey bee operations (mainly used for honey production). In June 2010, we visited all five honey bee operations on Newfoundland to visually evaluate colony strength and quantify arthropod parasites, and to collect samples to be assessed molecularly for viruses and *Nosema* spp. Because of variation in sizes of commercial operations, the number of colonies sampled per operation was 1, 4, 5, 10, and 35 colonies out of a possible 2, 4, 8, 19, and 82 colonies, respectively. In total, we sampled 55 of 115 (47.8%) of all colonies in Newfoundland. Commercially maintained honey bees are typically housed in wooden boxes with vertical frames that have wax honeycomb adhering to them. Frames can be removed for inspection of a series of variables that are assumed to be related to colony strength [37]. To assess colony strength, 10 or 20 frames per colony (depending on whether the colony had a single or double brood chamber) were assessed visually on both sides for each of the following on a 4-point scale (0 indicating absence or barely detectable, 1 indicating clearly detectable but <10% of a frame, 2 indicating between 10 and 50% of a frame, and 3 indicating >50% of a frame): occupied by adult bees, empty, containing honey, containing pollen, containing eggs, capped brood cells (containing developing pupae) , and uncapped brood cells (containing developing larvae). For each colony, scores were summed for both sides of all frames, and where only 10 frames were sampled, doubled to produce a standardized score per 40 sides of 20 frames (maximum possible score for any metric within a colony was hence 120).

During each frame assessment, bees were scanned visually for ∼1 min for signs of the following diseases. Deformed wing is a continuum of signs (image in [38]) that can occur (rarely) in the absence of deformed wing virus (DWV) [33], [39]. Briefly, one or both wings become stunted or curled in adults. K-wing is where wing morphology is normal, but wings are held asymmetrically over the back, with at least one hindwing uncoupled from its corresponding forewing and extending further forward across the lateral line than is normal (i.e. in front of forewing). Its cause is unknown. Chalkbrood is an infection of brood caused by the fungus *Ascosphaera apis* (Maasen ex Claussen); it occurs most commonly in spring or in damp weather. Brood becomes chalky, mummified pellets [40]. Foulbrood (American and European) is a highly contagious infection of brood caused by a few species of bacteria; the disease is associated with abnormal looking brood that may be discoloured. Foulbrood is associated with characteristic odours that facilitate diagnosis [40]–[42]. Finally, we also looked for small hive beetle (*Aethina tumida*) (Murray) and *Tropilaelaps* (Delfinado & Baker) spp. ectoparasitic mites.

### Qualitative assessments of viruses

To test for viruses, 23 colonies from the preceding sample were randomly chosen for molecular screening: three or fewer from the four smaller operations and 15 from the largest operation. Ten adult worker bees were collected from the broodnest, crushed using sterilized forceps, and placed in 1.5-ml tubes (5 bees/colony/tube; 2 tubes/colony) containing RNAlater (Qiagen). Tubes were stored at −80°C, except when kept in a cooler with dry ice during the collection period, or in a cooler with ice packs during transport from Acadia University to The Pennsylvania State University for molecular analyses. Total RNA from each sample was extracted using TRIzol (Invitrogen) and re-suspended in 20 µl of DEPC-treated water. Presence of five viruses was determined using RT-PCR analyses using primers for black queen cell virus (BQCV) (forward and reverse primers in Table 1), DWV, Israeli acute paralysis/Kashmir bee viruses (the same Israeli acute paralysis virus primer sequences were used to detect Kashmir bee virus; sequencing of PCR product is used to differentiate between the two if either are detected), and sacbrood virus [43]–[45]. For an internal control, 514 base pairs of the honey bee actin gene were amplified (Table 1). All primers with the exception of BQCV were selected from previously published sources due to their proven sensitivity and specificity to the target of interest over several years of use. BQCV primers were designed for this experiment using Primer 3 (Whitehead Institute for Biomedical Research) [46]. Trial runs on samples known to be positive or negative for BQCV were tested in order to determine the specificity of the primers. cDNA was synthesized using M-MLV reverse transcriptase (Promega) and PCR was carried out for actin and sacbrood virus using the following parameters: an initial denaturing for 8 min at 94°C and 35 cycles of 94°C for 55 s, 51.5°C for 55 s, and 7°C for 1 min 25 s, with a final extension step for 10 min at 72°C. For BQCV, DWV, and Israeli acute paralysis virus/Kashmir bee virus, PCR was carried out using the following parameters: an initial denaturing for 8 min at 94°C and 38 cycles of 94°C for 1 min, 55°C for 1 min and 72°C for 1 min 15 s, with a final extension step for 10 min at 72°C. A negative control lacking template DNA and a positive cDNA control were included in the PCR reaction. Five µL of each RT-PCR product were electophoresed in a 1.5% agarose gel, stained with SYBR Safe DNA gel stain (Invitrogen), and imaged using a Gel Doc XR (BIO-RAD).

**Table 1:** Primers used for virus diagnosis.

Target	Forward primer	Reverse primer	Reference
Black queen cell virus	TGGCAACCTAGCCATTTAGC	GGTAGTGGGAGCTGACCAAA	This study
Deformed wing virus	CTCGTCATTTTGTCCCGACT	TGCAAAGATGCTGTCAAACC	[43]
Israeli acute paralysis/Kashmir bee viruses	GGTCCAAACCTCGAAATCAA	TTGGTCCGGATGTTAATGGT	[45]
Sacbrood virus	CACTCAACTTACACAAAAAC	CATTAACTACTCTCACTTTC	[60]
Actin gene	ATGAAGATCCTTACAGAAAG	TCTTGTTTAGAGATCCACAT	[44]
qDWV	CATGCATTACGTTTGGATGCA	TTCATCAGGAGCACAACCTACAG	This study
qActin	ATGCCAACAGTGTCCTTTCTGG	GACCCACCAATCCATACGGA	[20]

### Sequence analysis of DWV

Because false positives are common for DWV (S. Martin pers. comm.), positive samples had their PCR product identification confirmed by sequencing. PCR products were treated with ExoSAP-IT (USB) according to manufacturer's instructions and both strands were sequenced. Sequence data were aligned using MEGA 5.0 [47]. Nucleotide sequences for DWV determined in this study were deposited in GenBank (Accession nos. KJ809605-KJ809621).

### Quantitative assessments of DWV using SYBR green RT-qPCR

All samples were analyzed for DWV using SYBR green chemistry as follows. PCR reactions contained 50 ng RNA, 2× Power SYBR Green PCR Master Mix (Applied Biosystems), and 1 µM of each primer. Primers are given in Table 1. qDWV was designed for this experiment using Primer Express software (Applied Biosystems) for Real-Time PCR, Version 3.0. Reactions were set up in 96-well reaction plates and run using a 7500 Fast Real-Time PCR System (Applied Biosystems) using cycling conditions of 50°C for 2 min, 95°C for 10 min followed by 40 cycles of 95°C for 15 sec and 60°C for 1 min. To ensure that a single product was amplified, and no contamination was present in the no-template controls, a final dissociation curve was produced.

### Quantitative assessments of *Nosema* spp

*Nosema* spp. spores have been detected in Newfoundland honey bees before [36], but molecular identification of species has not previously been done. A sample of 30 adult worker bees was collected from the broodnest of each colony and kept at −20°C, except during transport when they were kept in a cooler with dry ice. *Nosema* intensity (spores per bee) was estimated by crushing the 30 abdomens in 30 ml distilled water and examining resulting suspensions for spores using a haemocytometer and light microscope [48]. Molecular analyses [49] were performed on all spore suspensions using conventional duplex PCR with previously described primers 321APIS-FOR and 321APIS-REV for *N. apis* and 218MITOC-FOR and 218MITOC-REV for *N. ceranae* [11].

Molecular protocols were described previously in detail [49]. Briefly, total genomic DNA was obtained from each composite crushed bee suspension using an Ultra Clean Tissue DNA Extraction Kit (Mo Bio Laboratories). Additionally, two crushed bee samples with no observable spore counts were included as negative controls. Two biological replications of DNA isolation were included when required. DNA quality and quantity was measured with a Nanodrop 1000 spectrophotometer (Fisher Scientific). The 25-uL conventional PCR (two technical replications per biological replication) reaction mix consisted of 1× ID taq buffer and 1.0 U taq (ID labs Inc.), 400 nM of each primer, and 10 ng of DNA template. Negative (i.e. no template DNA) and positive (i.e. samples previously sequence-verified for *N. apis* or *N. ceranae* infection) controls were included. Amplification was achieved using a Biometra TGradient thermocycler (Montreal Biotech Inc.) using the following parameters: initial denaturing step of 94°C for 4 min followed by 35 cycles of 94°C for 15 s, 60°C for 30 s and 72°C for 45 s, and ending with a final extension of 72°C for 7 min. PCR products were separated by electrophoresis in 1.4% agarose gels stained with SYBR Safe DNA stain (Invitrogen), and visualized with a GelDoc2000 system (Bio-Rad).

### Qualitative assessment of mites

Tracheal mite prevalence (% colonies infested) was estimated for 100 randomly selected honey bees from the same 55 colonies that were visually inspected. Tracheal tubes were exposed by cutting between the head and prothoracic segment, and immediately posterior to the point of wing attachment. These thoracic disks were soaked in 7.5% potassium hydroxide for ∼6 h at 75°C, and then examined under a dissecting microscope (modified from [40]).

*Varroa destructor* intensity (mites per bee) was estimated by agitating for ∼3 min ∼100 bees per colony in a stainless steel strainer in a basin containing windshield washer fluid (−40°C formulation). The basin was lined with a cotton sheet. This detaches mites that can be easily counted, including *Tropilaelaps* mites [50].

### Statistical assessment of parasite associations and effects on colony strength

Statistical analyses were conducted in SAS 9.3 (SAS Institute). We log+1-transformed DWV viral titres and *Nosema* spore counts. To test for associations among signs of disease and parasites, we used General Linear Mixed Models (GLMMs) where a sign or parasite was a response variable, remaining signs and parasites were simultaneous explanatory variables, and operation was a random effect. We sequentially removed non-significant associations until only significant associations remained in the model [51]. We tested whether our scoring of multiple metrics of colony strength could with Principal Components Analysis (PCA) be reduced to a smaller number of variables. Briefly, this method condenses multiple variables into a reduced set of variables so that fewer statistical tests are conducted, limiting the number of potentially spurious significant results. We used the broken stick model [52] to evaluate whether Principal Component (PC) eigenvalues were greater than expected by chance, and retained only PCs that met this criterion. To assess whether diseases were associated with colony strength measures, we used a GLMM where colony strength was our response, signs and parasites were explanatory variables, and beekeeper operation was a random effect.

## Results

### General observations from visual assessments

**Figure 1:**
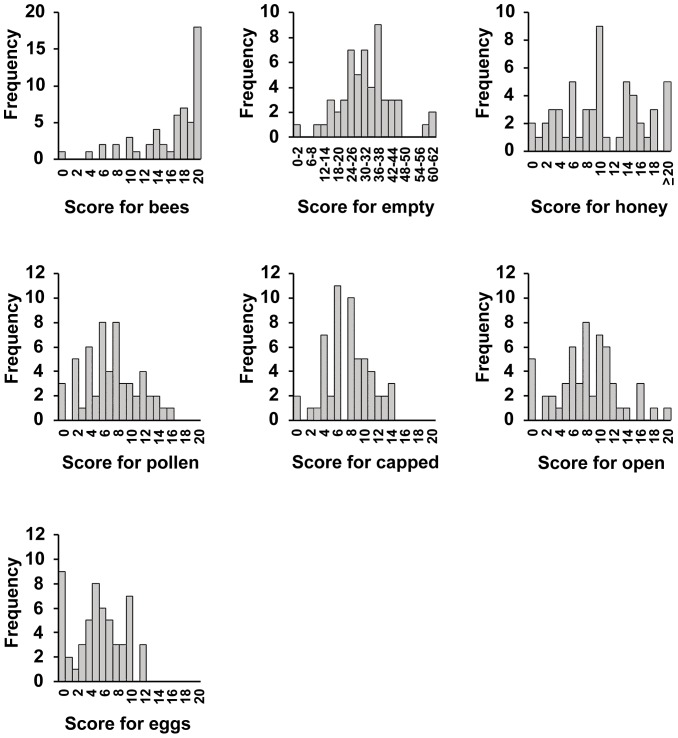
Frequency distributions of honey bee colony strength measures.

A total of 900 frames from 55 colonies on the island of Newfoundland was scored visually for metrics of colony strength (Fig. 1). Of 55 colonies sampled, none had deformed wings, 40 (73.7%) had K-wing, two (3.6%) had chalkbrood, none had either American or European foulbrood, and no small hive beetles were observed. From these visual assessments, we retained only K-wing visual assessment data for further analysis.

### Viruses

Of 23 colonies first screened for viruses, samples from one (5%) failed to amplify actin, suggesting that nucleic acids had degraded either before or during analysis. Of the remaining 22 colonies, none tested positive for the Israeli acute paralysis virus - Kashmir bee virus complex, none tested positive for sacbrood virus, and 16 (72.7%) had at least one sample positive for BQCV. Because of the potential for false positives, a second screening was performed for DWV using the same extracted RNA; actin was amplified in all of the second samples. In total, 22/23 colonies (95.7%) had at least one of four samples positive for DWV, and all 19 colonies on which sequencing was performed were confirmed to contain DWV viral titres (Table 2). Relative gene expression (compared to actin) ranged from 0.3 to 608.0 (Table 2).

**Table 2:** Results of PCR and subsequent sequencing of PCR products positive for deformed wing virus (DWV).

Operation	Sample	Positives	Gene expression relative to actin	
1	1	1		1
	2	4	1.3, 1.7	
	3	4	1.5, 2.6	
2	1	4	5.8, 10.6	
	2	2	70.4	2
3	1	2	2.7	2
	2	0	0.3	3
4	1	2	3.1	
	2	1		1
	3	2	338.3	
	4	2	0.6	2
	5	2	12.1	2
	6	4	1.8, 141.6	
	7	1	608.0	4
	8	4	2.1, 6.5	
	9	3	3.9, 6.2	5
	10	4	2.0, 9.9	
	11	3	13.9	6
	12	3	0.8, 3.8	6
	13	2	1.7	2
	14	1		1
	15	2	6.5	2
5	1	1		1

Two tubes were obtained from each colony for initial identification; these were each analysed twice for a total of four PCRs per colony. If both tubes were positive, relative gene expression is provided for both.

1 A DWV band was obtained in the earlier gel, but not in a later gel; possibly sample degradation. Sequencing not attempted.

2 One of two samples from the colony was positive for DWV in both analysis; DWV presence confirmed in second analysis.

3 RT-PCR was negative for DWV in both first and second gel but RT-qPCR showed very low levels of DWV. Sample was not sequenced; thus DWV presence was not confirmed.

4 No DWV bands were obtained in the first analysis, but one band was obtained in the second analysis, and presence was confirmed by sequencing.

5 In one of two sample, no DWV band was obtained in the first analysis, but a band was obtained in the second analysis, and presence was confirmed by sequencing. Second sample from this colony was positive both times.

6 DWV bands were obtained in the first gel for both colonies NF103 and NF112 but one of the two bands was faint for each colony; in the second analysis no band was observed from the sample with a faint band. The second analysis confirmed DWV was present (by sequencing) in both colonies.

### *Nosema* spp. assays

**Figure 2:**
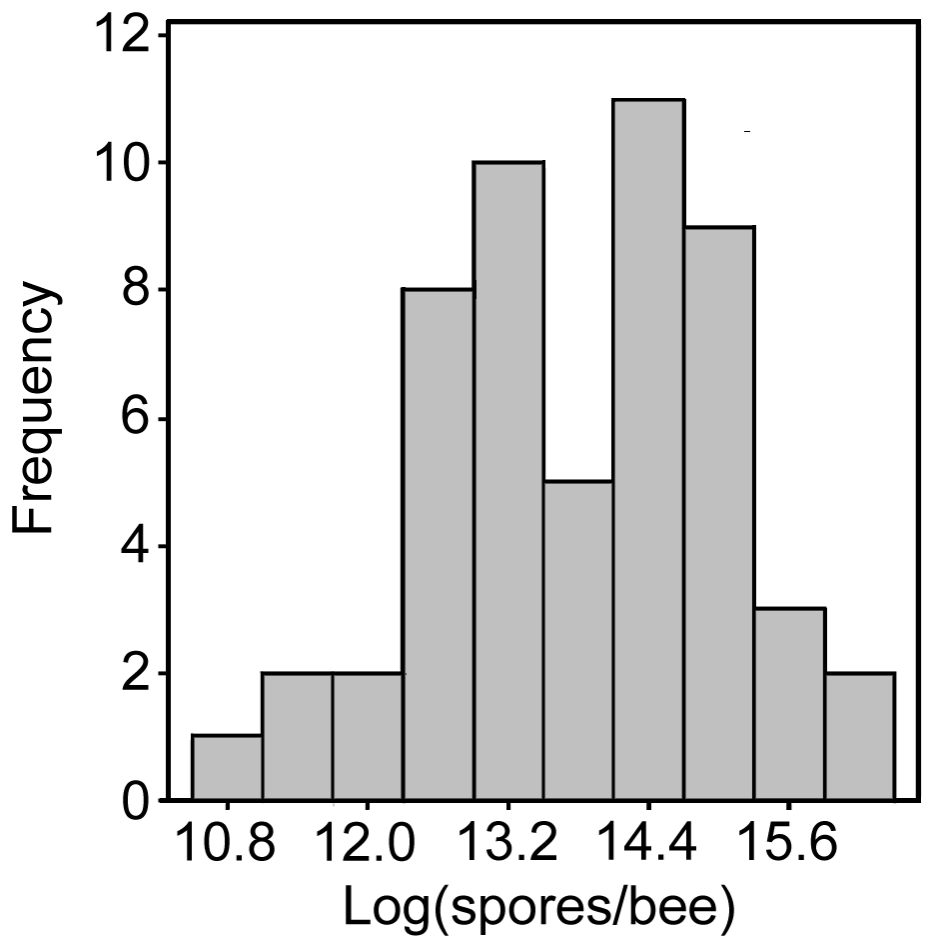
Distribution of *Nosema apis* infection intensities among honey bee colonies (excluding two colonies that were negative).

Of 55 colonies screened for *Nosema* spp. using light microscopy, 53 (96.4%) were infected. During an initial PCR run, *N. ceranae* was detected in 2 colonies from the large operation. When 30 different bees collected from those same colonies during the same sampling effort were analyzed, only *N. apis* was detected. We suspect those two initial samples may be contaminated because our laboratories have analyzed both *N. apis* and *N. ceranae* parasites; however, due to diligent lab hygiene, including appropriate PCR controls, we cannot rule out that we have detected early invasion of the parasite. As a precaution, we re-tested worker bee samples collected from those same hives on 1 October 2010, and again found *N. apis* only. Frequencies of intensities followed a negative binomial distribution that, after log+1-transformation, approximated a normal distribution (Fig. 2). Among infected bees, infection intensities ranged from 50,000 to over 13,000,000 spores/bee.

### Mite assessments

No *V. destructor*, tracheal, or *Tropilaelaps* spp. mites were detected.

### Associations among signs of disease, parasites, and colony strength

The sign of K-wing was positively associated with BQCV, and DWV titres were negatively associated with BQCV (Table 3). Remaining sign-parasite associations were not significant (Table 3).

**Table 3:** Associations among signs of disease and parasites.

	Explanatory variable			
Response variable	K-wing	BQCV	log(DWV titre+1)	log(*Nosema*+1)
K-wing	NE	+4.5*		
BQCV	+6.6*	NE	−8.6**	
log(DWV titre+1)		−9.1**	NE	
log(*Nosema*+1)				NE

Shown are *F*-statistics for variables left in General Linear Mixed Models (with operation as a random effect) after sequential removal of non-significant associations. Signs indicate whether associations were antagonistic (−) or synergistic (+).

*P<0.05,

**P<0.01.

NE denotes not entered in saturated model.

**Figure 3:**
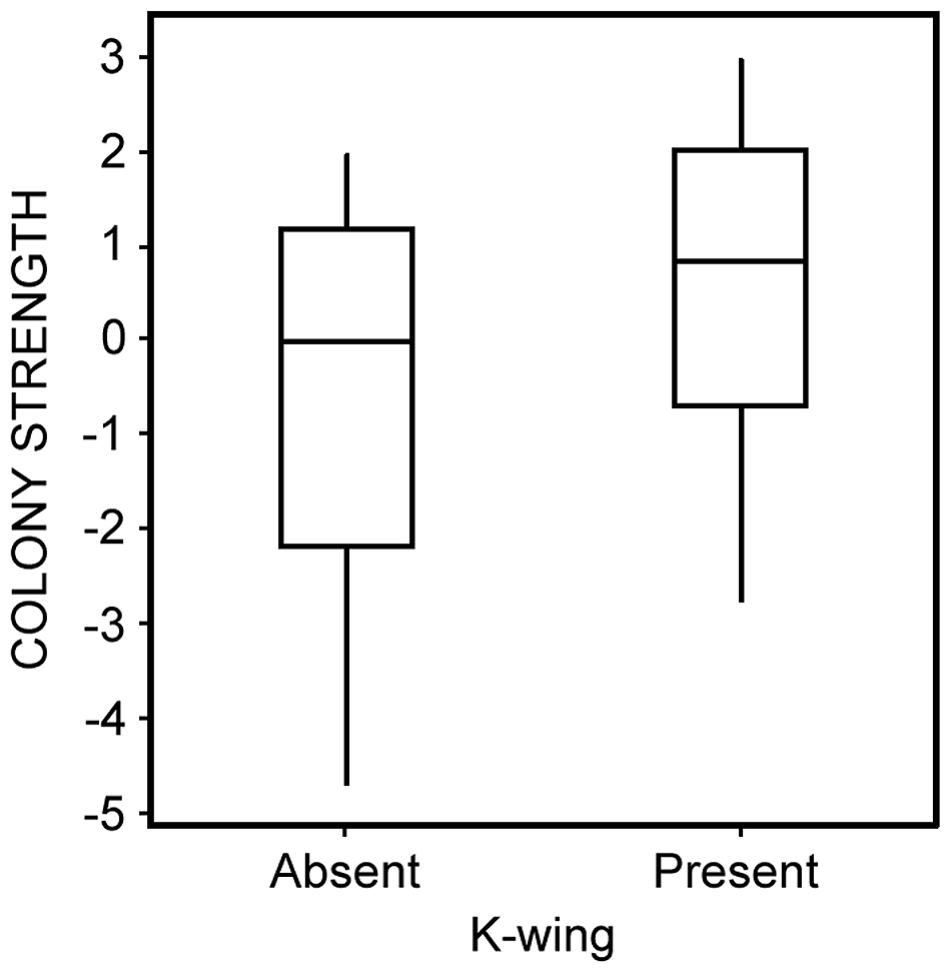
Relationships between COLONY STRENGTH (scores from a first PC from a PCA of seven measures; see Fig. 1) and K-wing. Shown are interquartile range (box), median (horizontal line within interquartile range), and data range (vertical lines). Statistics are reported in the text.

The first PC (hereafter COLONY STRENGTH) of our seven colony strength measures (Fig. 1) explained 53% of the total variation in those measures (null expectation is 37%); remaining PCs explained less variation than expected by chance and were not analyzed further. All variables in the PCA had fairly strong loadings (all ≥|0.30|; only “empty” had a negative loading of −0.40) on COLONY STRENGTH; we interpret this as indicating that healthier colonies had higher COLONY STRENGTH scores. After sequential removal of non-significant associations, only K-wing (*F*_1,16_ = 4.5, *P* = 0.05) remained associated (positively) with COLONY STRENGTH (Fig. 3).

## Discussion

Our visual and molecular screening adds Israeli acute paralysis virus, Kashmir bee virus, sacbrood virus, *N. ceranae*, small hive beetle, and *Tropilaelaps* spp. to the list of diseases that are apparently absent from Newfoundland's honey bees [36]; the proportion of the population we sampled bolsters our confidence in these results. The absence of *N. ceranae* and other parasites may also mean that fewer signs of other diseases will be detected [23], [25], [52], but see [53], [54]. From a single beekeeper's reports, colony losses were 3/23 (13.0%) over the winter of 2009–2010, and 2/16 (12.5%) over the winter of 2010–2011; these rates of mortality are similar to those reported for the United States before introduction of *V. destructor* [15]. Collectively, the number of parasite species missing from Newfoundland is significant for a variety of reasons. For example, Newfoundland bee colonies may represent a throwback to historical conditions of honey bee colonies and a yardstick against which operations in other parts of the world may be compared to gauge the collective effects of multiple invasive parasites. Because of the serious economic implications of these multiple diseases, Newfoundland has wisely restricted importation of honey bees [56]. This also means that honey and other products from Newfoundland will be relatively free of the multiple pesticides that are used to reduce parasites [57], and consequently these pesticides will not appear in the environment.

Although we did not detect chalkbrood or American or European foulbroods during our screenings, these parasites have been detected in previous surveys [36]. Our detections of BQCV and DWV are firsts for Newfoundland. As expected, the quantity of DWV (relative to actin) found in most of the pooled bee samples was low, which reflects the status of DWV prior to the introduction of *V. destructor*. However, several samples (e.g., NF018, NF063, and NF064) contained relative DWV quantities ranging from 117 to 608. These high DWV quantities, in the absence of *V. destructor*, are interesting. DWV, while non-virulent except when *V. destructor* mites horizontally vector it, is suspected to occasionally be the cause of colony death [58]. The high titres we observed may reflect occasional instances of DWV sufficient to cause colony death. As shown by Sumpter and Martin [59], DWV in the presence of *V. destructor* is more likely to lead to an epidemic of the virus within a colony and colony death. For populations free of *V. destructor*, the number of samples with detectable quantities of DWV was higher in our study of 23 Newfoundland colonies (59% of bees and 83% of colonies) as compared to 3 colonies from Sweden (40% of bees and 100% of colonies) [58]. Differences in the percent of bees positive for DWV may be due to year-to-year variations, time of year (high levels might be expected in late summer as opposed to late spring), or differences in the prevalence of the virus between the two populations. These reasons for differences could be important to explore. Fortunately, DWV and BQCV were not associated with our measures of colony strength, or with visual detection of deformed wing. These results are consistent with reports that these viruses are relatively benign in the absence of *V. destructor* mites [34], [35]. We detected a negative relationship between BQCV and DWV, but not with viruses and *N. apis*; antagonisms and synergies between BQCV and *Nosema* spp. have been reported in some studies [23], [53] but not others [54], [55]. The only common sign we detected visually was K-wing, but contrary to conventional expectation, it was associated with overall stronger colonies. This result may not genuinely suggest that K-wing is a sign of good health; it may instead be a function of variation among honey bee operations or spatial variation in distribution of the conditions or agents that are responsible for the sign.

Honey bee colonies within each operation are not entirely independent; unfortunately there are few operations in Newfoundland. Variation among operations may be substantial for a variety of reasons, including management intensity, density of colonies, etc. Most of our data came from a single large operation, and so our results may be biased to revealing relationships within that operation, although we did control for operation in our analyses.

Globally, it has become problematic to obtain parasite-free honey bees, whether for commercial or experimental purposes. In fact, the honey bee industry, including Newfoundland, has for several years relied on importation of queens from Australia and Hawaii [36]). We recommend continued restrictions on honey bee imports to Newfoundland.

In sum, on a globe where homogenization of honey bee parasites appears well-established, Newfoundland remains free from many of them. As a consequence, Newfoundland beekeepers incur economic savings with respect to the use of pesticides and potential economic benefits of a more organic set of products. The reduced parasite community in Newfoundland honey bees means that there are commercial and research opportunities unavailable in most parts of the world. The absence of *V. destructor* and *N. ceranae* in Newfoundland provides interesting populations for studies of honey bee epidemiology; however, we cannot state with absolute certainty that *N. ceranae* is absent in Newfoundland honey bees due to our initial positive detection in two colonies. Constant vigilance is therefore required. If *V. destructor* or *N. ceranae* are introduced to Newfoundland honey bees, there will be an opportunity test the conclusion reported by Martin et al. [35] on epidemiology of DWV following introduction of *V. destructor* to Hawaii. In Newfoundland, there may be an opportunity to study disease dynamics of *N. ceranae* without *V. destructor* or *V. destructor* without *N. ceranae*. Another area of possible research with Newfoundland bees is the exploration of consequences of a reduced number of stressors. *Varroa destructor* and *N. ceranae* are only two stressor not present in Newfoundland honey bee colonies. Moreover, these colonies are not transported long distances, queens are not imported from areas with *V. destructor*, and nutritional quality (nectar and pollen forage) available is high.
